# The effect of post-discharge education provided *via* telenursing on the quality of life and functional independence levels of elderly patients undergoing hip fracture surgery: a randomized controlled trial

**DOI:** 10.1093/her/cyag012

**Published:** 2026-04-29

**Authors:** Esra Üçpunar, Hasret Yalçınöz Baysal

**Affiliations:** Institute of Health Sciences, Department of Public Health Nursing, Ataturk University, 25240, Erzurum, Türkiye; Department of Public Health Nursing, Ataturk University Faculty of Nursing, 25240, Erzurum, Türkiye

## Abstract

The aim of this study is to evaluate the effect of post-discharge education provided *via* tele-nursing on the quality of life and functional independence levels of elderly patients who have undergone surgery due to a hip fracture. This randomized controlled, pre-test/post-test, single-center experimental study included 30 patients in the intervention group and 30 in the control group, conducted between August and April 2023. The intervention consisted of three online video consultations, three telephone calls, and an educational booklet, while no intervention was applied to the control group. Data were collected using a Patient Identification Form, QoL scale, and FIM. Ethical approval, institutional permissions, and informed consent were obtained. In the intervention group, 56.7% of the patients were female, while 53.3% of the patients in the control group were female (*P* = .79). The average age in the intervention group was 75.80, compared to 75.87 in the control group (*P* = .97). After the education, the post-test scores of the vitality and mental health subscales of the QoL scale were higher in the intervention group than in the control group (*P* < .05). After the education, the intervention group had a higher total FIM score than the control group, mainly driven by improvements in self-care and transfer subscales (*P* < .05). Post-discharge education provided *via* tele-nursing to elderly patients undergoing surgery for hip fractures improves their quality of life and functional independence levels.

Trial Registration: ClinicalTrials.gov, NCT06257615.

## Introduction

Aging is a physiological process that causes both physiological and anatomical changes in every organ [1]. These changes influence individuals’ daily activities, habits, work life, dependency levels, and interactions with their surroundings [2]. The musculoskeletal system is among the most affected by these changes. Osteoporosis is one of the major musculoskeletal health problems in the elderly. The most critical consequence of osteoporosis is fractures, with hip fractures being the most common [3].

As the global population ages, the incidence of osteoporotic hip fractures increases in parallel, by ~1%–3% [4]. Although Turkey has a lower hip fracture rate than Europe, the number of cases has significantly increased in the past 20 years [5]. The relatively lower prevalence of hip fractures in Turkey may be due to the lack of multi-center and adequate follow-up studies, the absence of necessary research, or deficiencies in records/data [6].

The increasing number of patients with orthopaedic conditions places a significant burden on hospital-based healthcare resources. Due to the financial strain on both individuals and the national economy, there is a growing need to reduce healthcare costs, utilize accessible technological solutions, and provide additional services [7]. Telehealth services, including phone follow-ups, allow for close and daily monitoring of patients, which has contributed to reduced costs and improved clinical outcomes [8]. A study conducted on patients aged 60 and above who underwent surgery for hip fractures concluded that tele-rehabilitation, a subset of telehealth, can enhance health and functionality while reducing economic burdens [9]. Tele-nursing practices, particularly designed to provide home care and eliminate transportation challenges, offer significant benefits and advantages to the elderly [10].

Additionally, it has been noted that the use of the internet by orthopaedic patients to access information has increased over the years, with computer-assisted interventions and phone-based care contributing to patient satisfaction, reducing hospital stays, and providing a safe and promising alternative to traditional care [11]. A study that emphasized the importance of elderly patients receiving the necessary support after hip fracture surgery to regain their independence found that while elderly individuals believed in their ability to recover, they needed support and adequate information during the recovery period, and nurses were aware of their patients’ needs [12].

Today, computers and the internet are widely used by nurses in fulfilling their educational role, one of the most important responsibilities in providing quality care. Patient education is frequently used in tele-nursing applications, which integrate telecommunications networks into the nursing field, allowing uninterrupted nursing care and patient education for distant patients [13, 14].

In Turkey, no study has evaluated the combined effects of post-discharge education provided *via* tele-nursing on the quality of life and functional independence levels of patients who have undergone surgery for hip fractures. It is believed that this study will contribute to short- and long-term innovations and developments in nursing education and will improve the quality of life and functional independence of individuals who undergo hip fracture surgery. The aim of this study is to determine the effect of post-discharge education provided *via* tele-nursing on the quality of life and functional independence levels of patients who have undergone hip fracture surgery.

### Hypotheses of the study

H1_1_: The post-education mean quality of life scores of the intervention group differ from their pre-education scores.

H1_2_: The post-education mean quality of life scores of the intervention group differ from those of the control group.

H1_3_: The post-education mean functional independence scores of the intervention group differ from their pre-education scores.

H1_4_: The post-education mean functional independence scores of the intervention group differ from those of the control group.

## Materials and methods

### Type, population and sample of the study

This is a randomized controlled pre-test/post-test single-center experimental study. This study was registered in ClinicalTrials.gov (NCT06257615).

**Figure 1:**
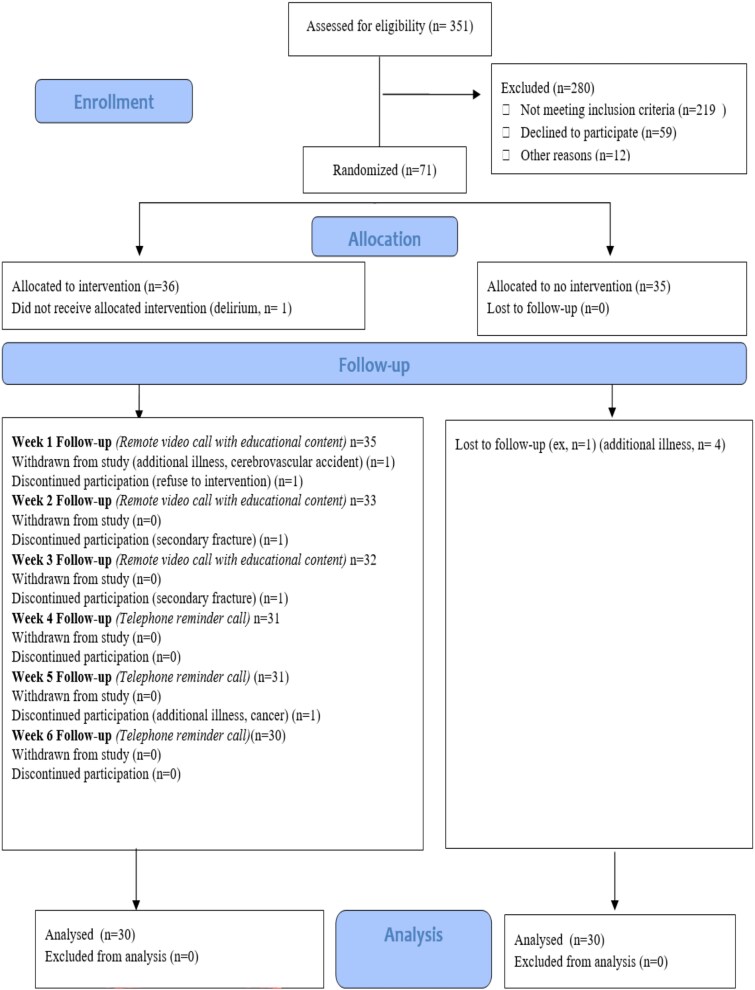
Consort diagram.

The study was conducted at the Orthopaedics and Traumatology Department of Istanbul Metin Sabancı Baltalimanı Bone Diseases Training and Research Hospital between March 25, 2021 and December 14, 2023. The study population consisted of patients who were operated on and discharged with a hip fracture diagnosis between August 13, 2022 and April 1, 2023. The sample comprised 60 patients who met the inclusion criteria and agreed to participate. The sample size was calculated based on the primary outcome, namely the change in quality of life and functional independence levels from baseline to six weeks after discharge. In line with randomized experimental studies comparing conventional care with telehealth interventions in patients who underwent hip fracture surgery, and particularly the study by Gilboa *et al.* [15], we assumed a large standardized mean difference (Cohen’s d) of ~1.19 between the intervention and control groups. Considering a two-sided type I error (α) of 0.05 and a minimum power (1–β) of 0.80, this effect size indicated that 30 patients per group would be sufficient. To account for possible dropouts during follow-up, we initially enrolled 36 patients in the intervention group and 35 patients in the control group (71 patients in total). During the data collection period, 11 patients withdrew for various medical and personal reasons, and the trial was completed with 60 patients (30 in the intervention group and 30 in the control group; Fig. 1, CONSORT diagram).

The post-hoc power of the study was calculated using the G^*^Power 3.1.9.2 program. Based on the data from 60 participants (30 in the intervention group and 30 in the control group), a standardized effect size (Cohen’s d) of 1.19 and a power of 0.99 at α = 0.05 were obtained. Although retrospective power analyses have limitations, this value suggests that the study had sufficient power to detect large between-group differences [16].

Patients aged 65 years and older who were hospitalized and operated on for a hip fracture due to a simple fall/self-level fall/osteoporotic fracture, were literate, had no vision or hearing impairments that would hinder communication, had no mental disorders (based on the patient’s own statement), had a primary caregiver aged 18 years or older who would cooperate during the study, and possessed the necessary technical equipment for online communication (phone, tablet, computer, *etc.*) were included in the study. Patients with a history of previous hip fracture surgery and those who were bedridden or using assistive devices for walking before the current fracture were excluded. The inclusion and exclusion criteria were determined based on verbal statements, patient records, and clinical observations.

### Randomisation & blinding

Patients were stratified based on two prognostic factors (age and gender) identified in randomized controlled trials in the literature and were evenly distributed into blocks [9, 15, 17, 18]. Within each stratum, participants were then randomly assigned to the intervention or control group using simple randomisation. Group allocation was performed by a trained surveyor who used a coin toss to determine assignment (heads = intervention, tails = control) within each block until the target numbers per group were reached. No central randomisation procedure was used. Although the stratification by age and gender was intended to balance important prognostic variables between groups, formal allocation concealment methods such as sealed opaque envelopes were not implemented, which may have introduced a potential risk of selection bias. Patients were divided into two age strata (65–74 years and 75 years and older) based on WHO guidelines (2015) [19]. After stratification, the groups’ homogeneity concerning control variables was evaluated and analysed.

Randomisation and group allocation were performed by a trained surveyor, and the tele-nursing intervention was delivered by the researcher to the intervention group. Because of the nature of the intervention, participants and the intervention nurse could not be blinded to group assignment. However, the pre-test and post-test data were collected by an independent trained surveyor using standardized procedures, and the statistician who analysed the data was blinded to group allocation [20].

To minimize the risk of expectation bias, the pre-test and post-test data (SF-36 and FIM scores) were collected by a trained surveyor who was not involved in delivering the tele-nursing intervention. While the nurse who provided the intervention was aware of group allocation, the surveyor followed a standardized protocol for data collection to ensure consistency. Because of the nature of the intervention, participants could not be blinded to their group assignment; however, the use of an independent surveyor for outcome assessment provided a level of blinding for the data collection process.

### Nursing intervention

The nursing intervention consisted of post-discharge education provided *via* tele-nursing for the intervention group. The educational content included three online video consultations (weeks 1, 2, and 3 post-discharge), three phone consultations (weeks 4, 5, and 6 post-discharge), and an educational booklet. All patients (both intervention and control groups) received routine care and discharge services from the doctors and nurses at the Orthopaedics and Traumatology Department of the hospital. This routine care included in-hospital education delivered by nurses and physicians prior to discharge, covering medication management (drug names, dosages, administration times, and potential side effects), wound care principles (dressing changes, signs of infection, and hygiene), follow-up appointments, and indications for seeking medical attention. This education was provided as a single, brief face-to-face session on the day of discharge, with no structured post-discharge follow-up calls, video consultations, or additional written/visual educational materials.

*Online Video Consultation:* Training sessions given as nursing interventions were conducted *via* video conference. The training began with introductions/greetings and a verbal assessment of the current health status. The training provided through online interview is structured according to the content of the prepared training booklet. To enhance interaction during the presentation of the training booklet, answer questions, and provide information about the process, and to ensure the comprehensibility and retention of the training, as well as to reduce the side effects of surgical operations, practical applications such as positioning, mobilization, and mechanical prophylaxis (exercise and compression stockings) were included. The patient and caregiver were asked to repeat these applications. At the beginning of subsequent video calls, the patients’ current health status was discussed by inquiring about how they were feeling, the presence of pain, the regularity of medication use, their daily exercise status, the control of pressure points, their nutrition and elimination status, body hygiene, and the regular use of compression stockings. The training continued when cooperative and compliant answers were received to these questions. Considering time management, the researcher concluded each session with a few easily understandable sentences summarizing the training content, and asked and answered questions from the patient and their relatives, especially to evaluate the effectiveness of the training. At the end of the video call, doctor’s appointments, if any, were reminded, and information about the next audio/video call was provided. The video consultation lasted an average of 20–25 min for each patient. However, the duration planning took into account the patient’s individual characteristics, health status, and the conditions during the training (readiness for training, charging time of technical equipment, unforeseen circumstances, *etc.*).

*Voice Telephone Calls:* Voice telephone calls were conducted three times in total using mobile phones: once in the fourth, fifth, and sixth weeks, following the completion of online video calls conducted within the first three weeks after the patients in the intervention group were discharged. The aim of these voice calls was to ensure the retention and continuity of the training provided during the online video calls, to continue supporting the recovery process, and to answer questions that might cause anxiety in both patients and caregivers regarding the illness but could be resolved remotely. The main topics discussed during the voice telephone calls included informing patients about their health status, answering questions from patients and their relatives about the illness and recovery process, guiding them with questions unrelated to the illness, supporting them to pay maximum attention to all aspects in the training booklet, especially exercise, dressing hygiene, and common adverse situations, and reminding them of both doctor’s appointments and their next appointment. Voice phone calls lasted an average of 5–10 min.

*Educational Booklet:* Patients in the intervention group were given an educational booklet prepared in line with the training content, enriched with visual content, and at an easy-to-read and understand level, so that they could easily follow and repeat the training along with the collection of pre-test data. Patients and caregivers were asked to read the booklet at least once. The content of the educational booklet includes osteoporosis, the disease process, correct positioning and exercises, things to know and pay attention to after surgery, unwanted but frequently encountered situations after surgery, contact information and follow-up examination time. Patients in the control group received the training booklet after the post-test data was collected.

### Outcomes

The primary outcome of the study was the change in health-related quality of life from baseline (pre-test) to six weeks after discharge (post-test). Health-related quality of life was assessed using the SF-36 Quality of Life Scale (QoL), which comprises eight subscales (physical functioning, role limitations due to physical problems, bodily pain, general health perception, vitality, social functioning, role limitations due to emotional problems, and mental health). Each subscale score ranges from 0 to 100, with higher scores indicating better health status and health-related quality of life.

The secondary outcome was the change in functional independence from baseline to six weeks after discharge, measured using the Functional Independence Measure (FIM). The FIM consists of 18 items grouped into motor and cognitive domains and yields subscale and total scores, with higher scores indicating greater independence.

For both the QoL and FIM measures, pre-test assessments were performed face-to-face during hospitalization before discharge, and post-test assessments were conducted six weeks after discharge. Between-group comparisons (intervention versus control) of post-test scores were performed using independent t-tests, while within-group changes from pre-test to post-test were analysed using paired t-tests. A *P* < .05 was considered statistically significant.

### Data collection tools

Data were collected using the ‘Patient Introduction Form,’ ‘Quality of Life Scale (QoL),’ and ‘Functional Independence Measure (FIM).’

*Patient Introduction Form.* This form, prepared by the researcher based on the literature and expert opinions, consisted of 15 questions: 10 questions assessing sociodemographic characteristics and five questions assessing disease-related information.

*Quality of life scale (QoL, SF-36).* The QoL scale, developed by Ware and Sherbourne to measure health-related quality of life, is a widely used tool that allows comprehensive measurement in multiple dimensions [21, 22]. The 36-item QoL scale includes eight subscales: ‘physical function, social function, physical role limitations, emotional role limitations, mental health, vitality, pain, and general health perception’ and 2 main dimensions: ‘physical and mental’ [21–24]. Each subscale is scored separately, and there is no total score. The higher the score (up to 100), the better the health status and health-related quality of life. Lower scores (0) indicate worse health status and reduced health-related quality of life. The Turkish validity and reliability study of the QoL scale was conducted by Koçyiğit *et al.* in 1999 with patients with rheumatoid arthritis, and the internal consistency coefficient was found to be between 0.73 and 0.76 [24].

*Functional independence measure.* The FIM was developed in 1986 to create a uniform data system in medical rehabilitation in the United States [25]. It assesses an individual’s degree of independence in physical and cognitive activities. The FIM consists of 18 items, measuring physical (motor) and cognitive functions. Physical function includes 13 items, while cognitive function includes five items. Each item is rated from 1 to 7, where 1 indicates total dependence and 7 indicates total independence. The national adaptation of the FIM was conducted by Küçükdeveci *et al.* in 2001 with patients with stroke and spinal cord injuries, with a Cronbach’s alpha coefficient of 0.81, demonstrating that the FIM is a valid and reliable tool [26]. In this study, the pre-test Cronbach’s alpha coefficient for the FIM was 0.881, while the post-test Cronbach’s alpha was 0.545.

### Data collection

Pre-test and post-test data were collected face to face at the hospital by a trained surveyor independent of the researchers, after obtaining written and verbal informed consent from the patients and their relatives. They were informed about how to answer the data collection form, the confidentiality of the data, and its purpose.

### Evaluation of data

The data obtained in the study were analysed using the free trial version of SPSS Statistics 25.0 (Statistical Package for Social Sciences) for Windows program. A *P* value of less than .05 was accepted as the significance level. Chi-square analysis was applied to test the homogeneity of the groups with respect to categorical baseline variables. For quantitative data with normal distribution, independent t-tests were used to compare the intervention and control groups, and paired t-tests were used to compare pre-test and post-test scores within each group. For the comparison of continuous outcomes between the intervention and control groups, point estimates of the absolute mean differences and their corresponding 95% confidence intervals (CIs) were calculated and reported in addition to *P*-values.

### Ethical principles of the research

The research ethics committee permission was obtained from the Clinical Research Ethics Committee of a university, and the institutional permission was obtained from the hospital where the research was conducted. Those who were willing to participate in the research were included in the research. Since individual rights must be protected in the research, the Helsinki Declaration of Human Rights was adhered to during the study.

## Results

When the demographic characteristics of the patients included in the study were examined, it was found that 56.7% of the patients in the intervention group and 53.3% of the patients in the control group were female (*P* = .79), and the mean age of the patients in the intervention group was 75.80, while the mean age of the patients in the control group was 75.87 (*P* = .97). The demographic variables of the patients in both the intervention and control groups, such as gender, marital status, education level, family type, social security, income status, and place of residence, were similar (*P* > .05) (Table 1). An independent t-test was applied to test the differences in age, height, weight, and BMI values between the groups, and no statistically significant differences were found between the groups (*P* > .05).

**Table 1:** Comparison of the socio-demographic characteristics of the patients by group.

**Socio-demographic characteristics**		**Intervention group**		**Control group**		**Test statistic**	***P-*value**
		***n***	**%**	***n***	**%**		
**Sex**	Female	17	56.7	16	53.3	0.067^**^	.795
	Male	13	43.3	14	46.7		
**Marital status**	Single	9	30.0	12	40.0	0.659^**^	.417
	Married	21	70.0	18	60.0		
**Education level**	Primary school	13	43.3	19	63.3	2.473^**^	.290
	Middle school	7	23.3	4	13.3		
	High school	10	33.3	7	23.3		
**Family type**	Nuclear family	16	53.3	21	70.0	1.763^**^	.184
	Extended family	14	46.7	9	30.0		
**Social security status**	Yes	29	96.7	28	93.3	0.351^**^	.554
	No	1	3.3	2	6.7		
**Income status**	Income less than expenses	4	13.3	4	13.3	0.315^**^	.854
	Income equal to expenses	12	40.0	10	33.3		
	Income greater than expenses	14	46.7	16	53.3		
**Place of residence**	Village	4	13.3	4	13.3	0.325^**^	.850
	District	11	36.7	9	30.0		
	City center	15	50.0	17	56.7		
**Total**		**30**	**100.0**	**30**	**100.0**		

Notes *n*: number; %: percentage, ^**^ chi square analysis.

The comparison of the QoL Scales of the patients by group is shown in Table 2. At six weeks after discharge, the intervention group had higher scores in the vitality and mental health subscales compared with the control group. The mean difference in vitality was 5.7 points (95% CI 0.4 to 11.0, *P* = .036), and the mean difference in mental health was 13.2 points (95% CI 7.5 to 18.9, *P* < .001). A statistically significant difference was found between the pre-intervention and post-intervention test mean scores across all subscales of the QoL scale for the patients in the intervention group (*P* < .05).

**Table 2:** Intra-group and inter-group comparison of patients’ pre-test and post-test mean scores according to the sub-scales of QOL scales.

**QOL sub-scales**	**Group**	**Pre-test Mean ± SD**	**Post-test Mean ± SD**	**Within-group *P***	**Between-group Mean Diff (95% CI)**	**Between-group *P***
**Physical function**	Intervention	7.33 ± 8.68	33.00 ± 16.90	.000^**^	2.8 (95%CI:-5.7 to 11.4)	.513^*^
	Control	4.50 ± 8.24	30.17 ± 16.43	.000^**^		
**Physical role limitation**	Intervention	16.67 ± 23.06	44.17 ± 31.95	.000^**^	8.3 (95%CI:-9.2 to 25.8)	.345^*^
	Control	15.00 ± 21.38	35.83 ± 35.77	.000^**^		
**Pain**	Intervention	49.92 ± 18.15	61.58 ± 16.37	.003^**^	5.9 (95% CI: −2.8 to 14.6)	.180^*^
	Control	47.17 ± 14.27	55.67 ± 17.36	.028^**^		
**General health perception**	Intervention	41.11 ± 18.53	46.53 ± 17.68	.004^**^	1.1 (95% CI: −7.7 to 9.9)	.802^*^
	Control	39.86 ± 18.10	45.42 ± 16.43	.010^**^		
**Vitality**	Intervention	33.17 ± 11.71	46.00 ± 9.23	.000^**^	5.7 (95% CI: 0.4 to 11.0)	.036^*^
	Control	33.83 ± 11.72	40.33 ± 11.14	.001^**^		
**Social function**	Intervention	31.67 ± 20.95	40.83 ± 16.72	.000^**^	3.8 (95% CI: −5.4 to 12.9)	.416^*^
	Control	33.75 ± 20.28	37.08 ± 18.71	.234^**^		
**Emotional role limitation**	Intervention	24.44 ± 31.48	40.00 ± 35.45	.002^**^	2.8 (95% CI: −2.7 to 8.3)	.317^*^
	Control	26.67 ± 32.04	31.11 ± 32.68	.514^**^		
**Mental health**	Intervention	61.87 ± 13.52	71.73 ± 10.66	.000^**^	13.2 (95% CI: 7.5 to 18.9)	.000^*^
	Control	58.53 ± 13.35	58.53 ± 11.53	1.000^**^		

Notes QOL: Quality of Life; SD: Standard Deviation; CI: Confidence Interval; *P*: probability value; ^*^Independent T-Test, ^**^Paired T-Test.

The comparison of the FIM subscales of the patients by group is shown in Table 3. At six weeks after discharge, the intervention group had a higher total FIM score than the control group (mean difference 9.9 points, 95% CI 2.6 to 17.2), mainly driven by improvements in self-care and transfer subscales.

**Table 3:** Intra-group and inter-group comparison of patients’ pre-test and post-test mean scores according to the sub-scales of FIM score.

**FIM scale and sub-dimensions**	**Group**	**Pre-test Mean ± SD**	**Post-test Mean ± SD**	**Within-group *P***	**Between-group Mean Diff (95% CI)**	**Between-group *P***
**Self-care**	Intervention	20.50 ± 3.59	36.03 ± 3.48	.000^**^	4.7 (95% CI: 2.4 to 7.0)	.000^*^
	Control	20.43 ± 3.77	31.33 ± 5.11	.000^**^		
**Sphincter control**	Intervention	13.03 ± 1.63	13.13 ± 1.38	.573^**^	0.0 (95% CI: −0.7 to 0.7)	1.000^*^
	Control	12.93 ± 1.55	13.13 ± 1.31	.206^**^		
**Transfer**	Intervention	8.03 ± 3.39	17.07 ± 2.48	.000^**^	2.5 (95% CI: 1.0 to 4.1)	.002^*^
	Control	8.20 ± 3.33	14.53 ± 3.36	.000^**^		
**Locomotion/mobility**	Intervention	4.17 ± 1.21	10.43 ± 2.19	.000^**^	0.7 (95% CI: −0.5 to 1.9)	.260^*^
	Control	4.37 ± 1.19	9.73 ± 2.56	.000^**^		
**Communication**	Intervention	11.80 ± 2.73	12.00 ± 2.63	.110^**^	0.0 (95% CI:-1.4 to 1.3)	.960^*^
	Control	11.93 ± 2.63	12.03 ± 2.55	.264^**^		
**Social cognition**	Intervention	15.60 ± 4.87	16.97 ± 3.41	.001^**^	2.0 (95% CI: 0.1 to 3.9)	.037^*^
	Control	15.40 ± 4.67	14.97 ± 3.82	.397^**^		
**Functional independence measure total**	Intervention	73.13 ± 12.77	105.63 ± 12.48	.000^**^	9.9 (95% CI: 2.6 to 17.2)	.009^*^
	Control	73.27 ± 12.48	95.73 ± 15.60	.000^**^		

Notes FIM: Functional Independence Measure; SD: Standard Deviation; CI: Confidence Interval; *P*: probability value,^*^Independent T-Test, ^**^Paired T-Test.

## Discussion

The findings of this study, which aimed to evaluate the effect of post-discharge education provided *via* tele-nursing on the quality of life and functional independence levels of elderly patients operated on for hip fractures, were discussed in light of the hypotheses and the literature.

At the end of the tele-nursing education provided to elderly patients who had undergone hip fracture surgery, the quality of life of the patients was evaluated. In the comparison between the groups, the vitality and mental health dimensions of the patients in the intervention group increased significantly compared to the control group. After the intervention, the mean scores of all subscales, including physical function, physical role limitations, pain, general health perception, vitality, social function, emotional role limitations, and mental health, significantly increased in the intervention group compared to before the intervention (*P* < .05, Table 2). The increase in the mean scores of the QoL subscales in the intervention group can be explained by the comprehensive and repeated nursing interventions applied to the patient, the patient’s active participation in the process, and the early functional gains that positively reflected on the quality of life.

In the study, the increase in the post-test mean scores of physical function, physical role limitations, pain, and general health perception subscales in the control group compared to the pre-test scores was statistically significant (*P* < .05, Table 2). However, the greater increase in the intervention group compared to the control group highlights the impact of the intervention on increasing physical activity, general health perception, and vitality, as well as reducing physical role limitations and pain. The fact that the post-test mean quality of life scores were higher than the pre-test scores in both groups can be attributed to the reconstruction/healing of the hip joint and the improvement in pain and function, which positively reflected on the quality of life. This result is consistent with the findings of studies that have examined the effects of hip fracture and hip prosthesis surgery using different scales [27–32].

In the literature, no study has evaluated the effect of post-discharge education provided *via* tele-nursing on the quality of life of elderly patients operated on for hip fractures. Therefore, comparisons were made with similar studies. In a randomized clinical trial that investigated the effects of nursing interventions provided through telephone consultations on health status in patients aged 65 and over after total hip replacement, the control group received only standard conventional surgical treatment, while the intervention group received telephone-based counselling in addition to conventional surgical treatment at 2 and 10 weeks post-surgery. At the 3-month follow-up, while all patients showed improvement in health status, the intervention group showed significantly greater improvement in physical function, general health perception, and mental health compared to the control group [29]. The findings of this study are similar to the results of the current study. In our study, the fact that the intervention group showed significantly greater improvements in the vitality and mental health subscales compared to the control group suggests that structured, post-discharge telenursing support may be particularly effective in promoting psychological recovery and overall well-being in older adults after hip fracture surgery. Together with our results, these findings suggest that technology-supported follow-up after hip fracture may be a promising strategy to enhance both physical and mental components of recovery in older adults [31, 33].

Similarly, in Koçyiğit *et al.* study, which investigated the effect of home-based nursing interventions on the quality of life in elderly patients who had undergone hip fracture surgery, the post-intervention mean scores of the physical function, physical role limitations, pain, general health perception, and emotional role limitations subscales of the QoL scale were higher in the intervention group compared to the control group. However, no statistically significant differences were found in the mean scores of the vitality, social function, and mental health subscales between the intervention and control groups [30].

Similarly, in a study investigating the effect of post-operative discharge education on daily living activities and quality of life in patients who had undergone total hip replacement for any reason, it was found that one month after the operation, the mean QoL scores of the patients in the intervention group were higher than those in the control group. In the same study, statistical evaluation of the QoL subscales showed that the intervention group had significantly higher improvements in physical function, physical role limitations, emotional role limitations, and mental health compared to the control group (*P* < .05) [34].

In contrast, in the study by Szöts *et al.*, no significant increase was observed in the physical function, quality of life (measured by QoL), and general self-efficacy of patients with total knee arthroplasty who were followed up by telephone after discharge [35]. The intervention group, in addition to receiving standard care like the control group, was followed up by telephone on the 4th and 14th days post-discharge and received education on the most critical issues. The effect of the provided education was evaluated at 1 and 3 months, and no significant differences in QoL subscales were reported between the groups. Although there was short-term improvement in the physical function subscale and general self-efficacy scores during the first month, these improvements did not gain significance over time [35].

Based on the findings of this study, post-discharge education provided *via* tele-nursing to elderly patients operated on for hip fractures improved their quality of life. This result supports the study’s hypotheses H1/1, ‘The post-education mean quality of life scores of the intervention group differ from their pre-education scores,’ and H1/2, ‘The post-education mean quality of life scores of the intervention group differ from those of the control group.’

When the functional independence of elderly patients who had undergone hip fracture surgery was evaluated at the end of the post-discharge education provided *via* tele-nursing, it was found that the self-care, transfer, and social perception subscales, along with the total FIM scores of the patients in the intervention group, were significantly higher compared to the control group (*P* < .05, Table 3). After the intervention, the mean scores of the self-care, transfer, mobility, social perception subscales, and the total FIM score in the intervention group significantly increased compared to pre-intervention scores (*P* < .05, Table 3). These statistically significant results indicate that post-discharge education provided *via* tele-nursing was more effective in enhancing the independence of individuals in terms of self-care, transfer, mobility, and social perception. Moreover, the self-care, transfer, mobility subscales, and total FIM scores of the control group were also significantly higher in the post-test compared to the pre-test. This may indicate that the routine education provided in the clinic was effective for the patients in the control group. However, the greater increase in the intervention group highlights the importance of the intervention in enhancing functional independence in self-care, transfer, mobility, and social perception.

There are limited studies in the literature evaluating the effect of post-discharge telenursing training on functional independence levels in elderly patients who underwent surgery for hip fracture. Therefore, comparisons were made with similar studies. In their study, Ortiz-Pina *et al.* aimed to compare the effect of a multidisciplinary remote rehabilitation program on the functional recovery of older adults with hip fractures with in-person rehabilitation at home. They found that participants using the tele-rehabilitation program had higher Functional Independence Measurement scores compared to the control group [36]. The findings from that study are similar to the findings of the current study.

Similarly, in Şendir and Babadag’s study, which evaluated the effect of pre-operative education on post-operative physical adaptation and quality of life in patients undergoing total hip replacement, statistically significant differences were found between the intervention and control groups in the functional subscales of maximum walking distance, stair climbing, and transportation (*P* < .001). Additionally, significant improvements in the functional status, vitality, and general health perception subscales of the QoL scale were reported in the intervention group compared to the control group (*P* < .001) [27].

Based on these findings, post-discharge education provided *via* tele-nursing to elderly patients who had undergone hip fracture surgery increased the patients’ overall functional independence. This result supports the study’s hypotheses H1/3, ‘The post-education mean functional independence scores of the intervention group differ from their pre-education scores,’ and H1/4, ‘The post-education mean functional independence scores of the intervention group differ from those of the control group.’

Furthermore, a recent systematic review by Jansson *et al.* showed that computer- and telephone-delivered interventions in patients with orthopaedic conditions can improve patient outcomes and optimize the use of health-care resources [11]. Our study adds to this growing body of evidence by demonstrating that telenursing-based discharge education may specifically benefit quality of life and functional independence in older adults after hip fracture surgery.

As highlighted by Li *et al.*, careful evaluation of telehealth interventions requires a clear definition of outcomes, appropriate measurement tools and follow-up time points [32]. By using both the SF-36 and FIM at one-month follow-up, our study contributes outcome data that can inform the design and evaluation of future telenursing and telehealth programs for hip fracture patients.

### Limitations

This study used a convenience sample from a single centre and included only literate older adults with internet access and a dedicated caregiver, which limits the external validity of the findings. Central randomisation and sealed opaque envelopes were not used, so allocation concealment was not fully ensured and selection bias at the time of group assignment cannot be completely excluded.

Although pre-test and post-test data were collected by an independent trained surveyor, the study relied on self-reported SF-36 and FIM scores, and participants were aware of their group assignment; therefore, some degree of expectation bias may remain. In addition, we performed multiple comparisons across several SF-36 and FIM subscales without formal adjustment for multiplicity, which increases the risk of type I error and warrants cautious interpretation of *P*-values for individual subscales. Finally, the six-week follow-up period mainly reflects early recovery. While this is a clinically critical phase and our results demonstrate short-term benefits of tele-nursing, long-term outcomes such as re-fractures, falls, or readmissions were not assessed and should be addressed in future studies with extended follow-up.

## Conclusion

Post-discharge education provided *via* tele-nursing to elderly patients who had undergone hip fracture surgery improves their quality of life and functional independence levels.

Education provided through tele-nursing ensures the continuity of patient education regardless of distance, delivers ready and critical information tailored to the needs of patients who have undergone hip fracture surgery, and provides counselling and support to enhance their quality of life and functional independence, leading to positive patient outcomes.

In light of these results, it is recommended that post-discharge education be provided *via* tele-nursing to elderly patients who have undergone hip fracture surgery, taking into account their specific needs during the discharge process. It is also recommended that tele-nursing be adopted as an independent and standardized nursing practice in clinical and field applications. Further evaluation of the impact of tele-nursing education on the quality of life and functional independence of patients should be conducted through longer-term, larger sample size, cost-effective, multi-center, evidence-based studies.

## Data Availability

The data sets used and/or analysed during the current study are available from the corresponding author on reasonable request.
